# Tree-ring-based seasonal temperature reconstructions and ecological implications of recent warming on oak forest health in the Zagros Mountains, Iran

**DOI:** 10.1007/s00484-022-02380-5

**Published:** 2022-10-10

**Authors:** Mohsen Arsalani, Jussi Grießinger, Achim Bräuning

**Affiliations:** Institute of Geography, Friedrich-Alexander-Universität Erlangen-Nürnberg (FAU), Wetterkreuz 15, 91058 Erlangen, Germany

**Keywords:** Tree-ring width, Temperature reconstruction, Earlywood width, Zagros oak woodland, Mediterranean cypress, Insect outbreak

## Abstract

Abrupt changes in temperature have especially strong impacts on fragile ecosystems located in semi-arid regions. In this study, we analyzed tree-ring widths (TRW) of Mediterranean cypress (*Cupressus sempervirens* var. *horizontalis*) in the Zagros Mountains, Iran. Furthermore, we separately measured earlywood width (EWW) and latewood width (LWW) of Persian oak (*Quercus brantii* Lindl.) to examine if intra-annual resolution of tree-ring parameters of *Q. brantii* tree rings can be used as high-resolution paleoclimate proxies. Climate-growth relationships revealed that mean monthly maximum temperatures (Tmax) are a dominant factor determining radial tree growth and negatively affect both oak and cypress in the Zagros Mountains. Accordingly, we reconstructed two different seasonal windows of past Tmax variability, namely, January–March and June–August over the periods 1860–2015 and 1560–2015, respectively. Regime shift detection identified twelve warm and nine cold significant regime shifts in our summer Tmax reconstructions. The longest hot summer period occurred in the twentieth century, and two warm regime shifts occurred in 1999 and 2008. The highest values of the warm summer regime shift index occurred in 2008, which coincided with fungal pathogen attacks and insect outbreak of the oak leaf roller moth (*Tortrix viridana* L.) in the Zagros oak woodlands. Interestingly, we found common warm and cold periods in historic climate variability between the summer and winter Tmax reconstructions. Warm and cold regime shifts occurred simultaneously from 1955 to 2015, and significant regional warm summer and winter regime shifts have occurred between 2008 and 2015. The winter and summer Tmax reconstructions show high spatial correlations with large areas in West Asia, North Africa, and the eastern Mediterranean region. Our results strengthen initial studies on past climate variability in Iran and contribute to an enhanced understanding of past temperature variability in West Asia.

**Supplementary information:**

The online version contains supplementary material available at 10.1007/s00484-022-02380-5.

## Introduction

Climate change and its negative impacts on local communities and regional ecosystems are among the major challenges of our time (IPCC 2021). Increasing frequency of extreme events and record-breaking high temperatures have already raised concerns about the negative impacts of climate change (Valavi et al. 2019) and have become a major issue of concern for decision makers. Based on a recent IPCC report (IPCC 2021), the global surface temperature was 1.09 °C higher in 2011–2020 than 1850–1900, with larger increases over land (1.59 °C) than over the oceans (0.88 °C). Additionally, based on temperature records from the US National Oceanic and Atmospheric Administration (NOAA), the year 2020 was determined as the earth’s second-warmest year, and seven of the ten warmest years on record have occurred since 2014.

Located in the transition zone between the westerly dominated temperate and subtropical zones, Iran is highly vulnerable to the negative impacts of temperature extremes. Based on Iran’s Metrological Organization (IRIMO), the country’s mean temperature has increased by 2 °C over the last decades (1968–2020), considerably higher than the global average. Instrumental climate data show rapidly increasing trends of both, maximum and minimum temperatures in Iran (Fallah-Ghalhari et al. 2019), and National Drought Warning and Monitoring Center (NDWMC) has reported a nationwide and unprecedented drought during the hydrological year (October–May) 2020–2021, where total precipitation has declined about 50% in comparison to the long-term mean (1950–2020). This rapid increase in temperatures has especially strong impacts on semi-arid climate zones with limited water resources. Extremely low temperatures during winter (Ghanghermeh et al. 2015) and extremely high temperatures during dry summer have both devastating impacts on the regional ecosystems. High temperatures intensify the negative impacts of severe droughts and lead to an increase in socio-economic and eco-physiological challenges for the semi-arid parts of the country. As related negative consequences of the abrupt changes in climate variables, oak dieback and insect outbreaks were frequently reported in the Zagros oak woodlands in recent years (Mirabolfathi 2013; Pourhashemi et al. 2017).

The catchments in the Zagros Mountain range are the main source of water supply for the surrounding hyper-arid regions. As an elevated heat source, it is a major driver in the formation of subtropical high-pressure cells over the Middle East in summer (Zaitchik et al. 2007). Intensified heavy rainfalls, enhanced evaporation from water bodies, reduction in winter snowfall, and the disappearance of permanent snow cover in the high mountain areas are some of the negative consequences caused by increasing temperatures in the region. These changes result in serious threats and damages to local communities and ecosystems. Especially, the increase in summer temperatures in the Zagros Mountains and surrounding arid areas results in an increased demand for irrigation water, coupled to enhanced competition for water sources between local communities. As a consequence, groundwater reserves are estimated to have been depleted by about 74 km^3^ during the persistent dry period lasting from 2002 to 2015 (Ashraf et al. 2021).

Summer maximum temperature is an important parameter for forest growth, strongly influencing photosynthetic activity and the carbon assimilation of trees (Gu et al. 2022). The lack of available instrumental climate records before the 1950s hampers analysis of long-term mean monthly maximum temperature (Tmax) variability in Iran. Due to the impacts of temperature variability on the dry forest biome, water sources, and sensitive ecosystems, seasonal reconstructions of Tmax are required to place recent trends seen in instrumental climate data in a long-term perspective. Tree rings, as an annually and even intra-annually resolved climate proxy, have been widely used to reconstruct temperature on regional (e.g. Trouet 2014; Chen et al. 2016; Huang et al. 2019; Lara et al. 2020; Zhu et al. 2021) as well as on hemispheric scales (e.g., Briffa et al. 2004; Anchukaitis et al. 2017; Büntgen et al. 2021). Although a few precipitation reconstructions using different tree-ring parameters of broad-leaved and coniferous trees are existing for Iran (Arsalani et al. 2018a, 2021; Foroozan et al. 2020; Azizi et al. 2013), knowledge on past temperature variability in the country remains scarce, and only one tree-ring-based temperature reconstruction for the Zagros Mountains is available (Arsalani et al. 2015). Moreover, the number of tree-ring-based temperature reconstructions in the surrounding countries is also low (Heinrich et al. 2013; Köse et al. 2017); thus, it is important to reconstruct temperature histories for the Zagros Mountains to examine past regional temperature variability and the climate forcing mechanisms at work. Despite the proven potential of oak tree rings to serve as a climate proxy, earlywood and latewood series derived from this species have not yet been used separately to reconstruct seasonal aspects of climate history in Iran. The presence of old coniferous trees in the southern Zagros Mountains and the high potential of oak trees for intra-annual climate reconstructions (Arsalani et al. 2018b) make the Zagros Mountains highly suitable for dendroclimatic studies.

The main objectives of this study are (1) to investigate how *Quercus brantii* and *Cupressus sempervirens* trees located in the Zagros Mountains respond to the seasonal variability of maximum and minimum temperatures; (2) to reconstruct past seasonal variability of Tmax using annually and intra-annually resolved tree-ring series of *C. sempervirens* and *Q. brantii*; (3) to identify the forcing mechanisms responsible for the variations in seasonal Tmax in the semi-arid region of Iran, including climate regime shifts; and (4) to investigate related influence of climate change on the regional oak decline and insect outbreaks in oak woodlands in the Zagros Mountains.

## Material and methods

### Study area and climatic conditions

The northwest to southeast orientated Zagros Mountain range in Iran is a part of the Fertile Crescent. The mountainous region serves as a water tower for the surrounding arid and semi-arid basins and has significant impacts on atmospheric circulation through physical and thermal dynamic effects over the Middle East (Zaitchik et al. 2007; Zarrin et al. 2011). Rich pastures and permanent water sources have made the Zagros Mountains forest-steppe ecoregion one of the main areas for pastoral nomadism activities in Iran. Traditional pastoralism and dry farming in the Zagros Mountains are highly adapted to the seasonal precipitation pattern, showing a precipitation maximum during November to April. The Zagros Mountains represent 40% of Iran’s forests (Sagheb-Talebi et al. 2014), with Persian oak (*Quercus brantii* Lindl.) as the dominant tree species. *Q. brantii* is a native ring-porous species in the Zagros Mountains growing at elevations between 700 and 2300 m a.s.l. At higher elevations, oak forests are replaced by a more open forest type composed of more drought-tolerant tree species. In the southern Zagros Mountains, these forest steppes are locally mixed with *Cupressus sempervirens* var. *horizontalis* (Mill.), an evergreen coniferous species growing in small colonies in some valleys, between 1220 and 2000 m a.s.l. in Tang-Soulak site.

**Fig. 1:**
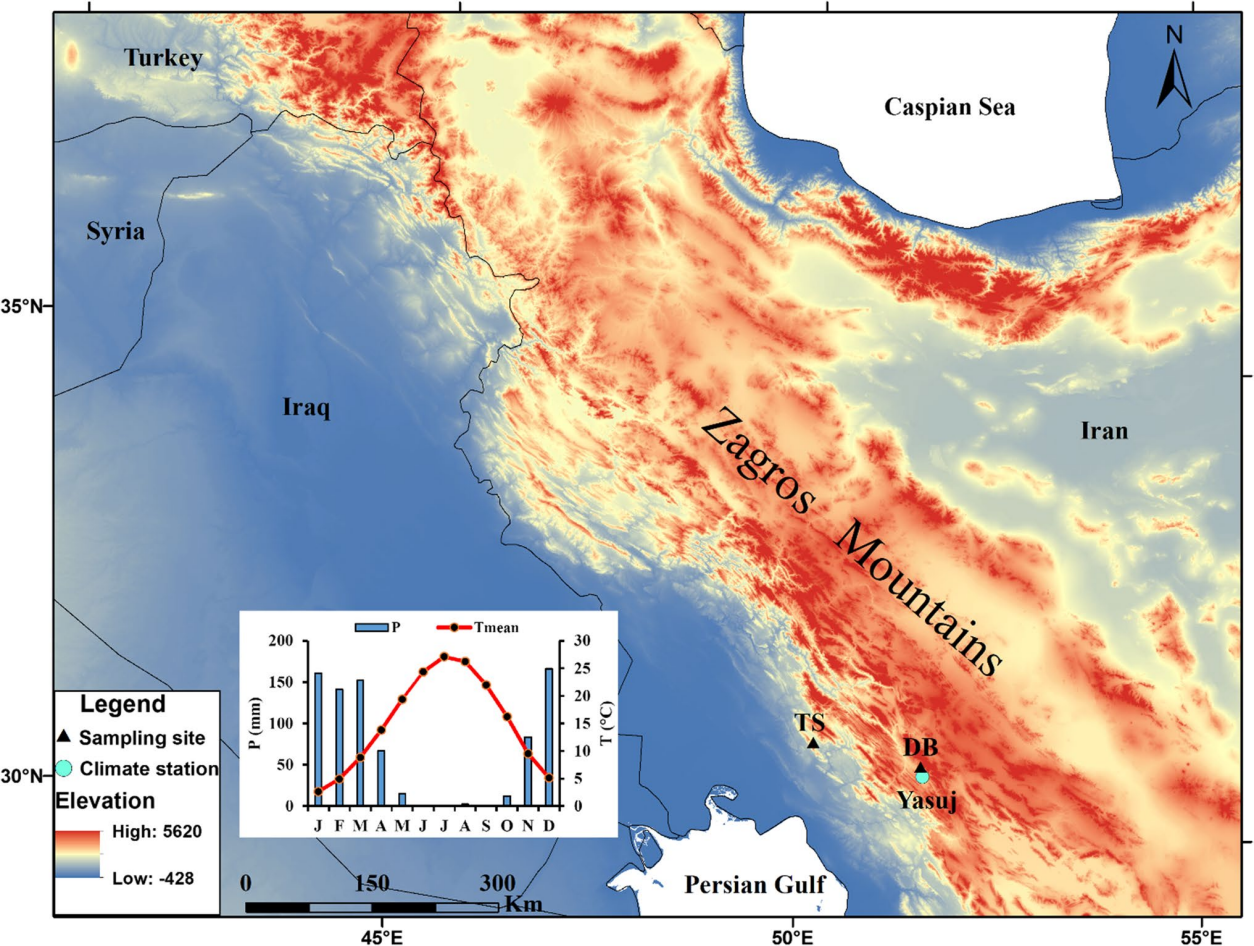
Location map of sampling sites and Yasuj climate station in the Zagros Mountains, Iran. The inserted climate diagram shows monthly precipitation and mean monthly temperatures of Yasuj meteorological station (1816 m a.s.l.) for the period 1987–2019

Our sampling sites Deh-Braftab (DB; 30°46′67″ N, 51°30′87″ E; 1810 m a.s.l.) and Tang-Soulak (TS; 30°59′12 N, 50°09′01″ E; 1587 m a.s.l.) are located in the southern parts of the Zagros Mountains (Fig. 1), and regional climate is mainly affected by the interplay of the mid-latitude Westerlies and the subtropical high-pressure cell (STHP). This interplay results in a pronounced seasonality with cold-humid (October–May) and warm and dry (June–September) periods (Fig. 1); therefore, both study sites are prone to mainly dry conditions during June to September, and the region’s highest and lowest temperatures occur in July (27 °C) and January (3.5 °C), respectively.

### Sample collection and chronology development

We collected 24 cores from 18 *Q. brantii* trees in DB site and 19 cores from 17 *C. sempervirens* trees in TS sites (Fig. 1, Table 1). Old trees with no obvious damage on the stem and canopy were sampled at breast height with an increment borer. Earlywood width (hereafter DB-EWW) and latewood width (hereafter DB-LWW) of *Q. brantii* were measured separately to obtain intra-annual climate signals from the intra-annual tree-ring parameters and color and vessel size differences were used as indicators to determine earlywood and latewood boundaries (Fig. S1a). Since latewood in *C. sempervirens* is represented by only a very thin band of latewood tracheids (Fig. S1b), we only measured total ring-width (hereafter TS-TRW) of this species.

DB-EWW and DB-LWW and TS-TRW were measured from bark to pith using a LINTAB 6 measuring system (Rinntech, Heidelberg, Germany) with a precision of 0.01 mm coupled to the analytical software TSAP-Win (Rinn 2003). Ring-width time series were cross-dated visually (Stokes and Smiley 1968) and statistically (Fritts 1976; Cook and Kairiukstis 1990) using the software TSAP-Win (Rinn 2003) and COFECHA (Holmes 1983). To remove non-climatic trends, raw ring-width series were standardized using the program ARSTAN (Cook and Holmes 1986). Detrending was performed using a cubic smoothing spline with a 67% frequency response cut-off to each series, which preserves 50% of the variance at a frequency equal to two-thirds of the length of each series. The individual detrended tree-ring series were averaged to mean site chronologies by computing the biweight robust mean (Cook and Kairiukstis 1990). Variance stabilization of each chronology was applied using the Keith Briffa’s RBAR-weighted method implemented in ARSTAN program (Cook and Krusic 2005). We tested several standardization methods and examined the climate growth patterns of different chronology versions produced by ARSTAN, namely, the standard chronology (STD), the residual chronology (RES), which is free of autocorrelation by the application of autoregressive modeling, and the Arstan chronology (ARS), which is generated by reincorporating the pooled autoregressive model into the residual chronology (Cook and Holmes 1986). Since we found the highest correlation between the standard chronology and climate data, this chronology was selected for further analyses.

Expressed population signal (EPS; Wigley et al. 1984) and inter-series correlation (Rbar) were used to assess the reliability of the site chronologies. Both Rbar and EPS were computed for 50-year moving windows with 25 overlap. Time series characteristics of the final chronologies were evaluated (Table 1) by computing the respective parameters first-order autocorrelation (AC1), standard deviation (SD), mean sensitivity (MS), and signal-to-noise ratio (SNR).

### Seasonal Tmax reconstruction, regional representativeness, and regime shift detection

Mean monthly maximum (Tmax) and minimum (Tmin) temperatures and monthly precipitation (P) of the Yasuj meteorological station (30°41′58″ N, 51°33′15″ E; 1816 m a.s.l.) were used to calculate climate-growth relationships. As analytical time windows, we investigated Tmax and Tmin monthly data from January of the previous growing year until September of the current year, as well as different seasonal windows. Monthly precipitation from October of the previous growing year to May of the current year was investigated since the region only receives precipitation during this period.

Based on the highest negative correlations between DB-EWW (*r* =  − 0.68, *p* < 0.01) and TS-TRW (*r* =  − 0.59, *p* < 0.01) chronologies and January–March Tmax (hereafter DB-JFM) and June–August Tmax (hereafter TS-JJA), seasonal Tmax were reconstructed using linear regression models. We tested the autocorrelation of climate data prior to developing the regression models. Due to the short length of the available instrumental climate data series (1987–2015), the leave-one-out cross-validation method (Michaelsen 1987) was used to evaluate the stability and reliability of the regression models (Fritts 1976). The stability of the regression models was assessed using the reduction of error (RE), the prediction residual error sum of squares (PRESS), the sign test (ST), and correlation coefficients statistics. Any positive value of RE indicates the validity of the regression model (Cook et al. 1994), and the ST shows the number of agreements and disagreements between the observed and the estimated data (Fritts 1976).

Spatial correlations between the reconstructed DB-JFM and TS-JJA and self-calibrated 0.5° × 0.5° regional gridded Tmax (CRU TS4.04; Jones and Harris 2008) time series (1980–2015) were calculated using the KNMI-Climate Explorer (https://climexp.knmi.nl/start.cgi) to assess the regional significance and the representativeness of the seasonal Tmax reconstructions. We also conducted climate regime shift analysis using the Shift Detection Program version 3.2 (www. beringclimate.noaa.gov/regimes/) to identify abrupt regime shifts in the summer and winter Tmax reconstructions and to characterize the patterns of Tmax fluctuations in the seasonal reconstructions. The Shift Detection Program is based on a sequential Student’s *t*-test calculated for a 10-year window moving over the mean values of the reconstructions (Rodionov 2004).

We calculated correlations between the reconstructed DB-JFM and TS-JJA and the North Atlantic Oscillation (NAO; Jones et al. 1997), the Atlantic Multidecadal Oscillation (AMO; Hansen et al. 2010), the Arctic Oscillation (AO; https://www.cpc.ncep.noaa.gov/products/precip/CWlink/daily_ao_index/ao.shtml), the Pacific Decadal Oscillation (PDO; Kennedy et al. 2011), and the Summer North Atlantic Oscillation (SNAO; http://www.psl.noaa.gov/data/gridded/data.ncep.reanalysis.derived.html) to investigate the influence of atmospheric circulation patterns on seasonal Tmax in the Zagros Mountains. NAO is a large-scale atmospheric pressure seesaw over the North Atlantic Ocean which is based on the surface sea-level pressure difference between the subtropical high and the subpolar low and has a strong effect on winter weather in Europe, Greenland, and northern Asia. SNAO represents the strength of a spatial pattern of pressure variability in the summer (July–August) over the northern North Atlantic Ocean. AO is a shifting of atmospheric pressure between the Arctic and the mid-latitudes of the North Pacific and North Atlantic, and positive and negative AO phases can affect temperatures in mid-latitudes. AMO is a periodically variation in the sea surface temperature (SST) of the North Atlantic Ocean that may last for several decades, and it is based on the average anomalies of SST in the North Atlantic Ocean with cool and warm phases. PDO is a long-term fluctuation of SST over the Northeastern Pacific Ocean, and its positive and negative phases can influence the atmospheric circulation patterns.

## Results

**Fig. 2:**
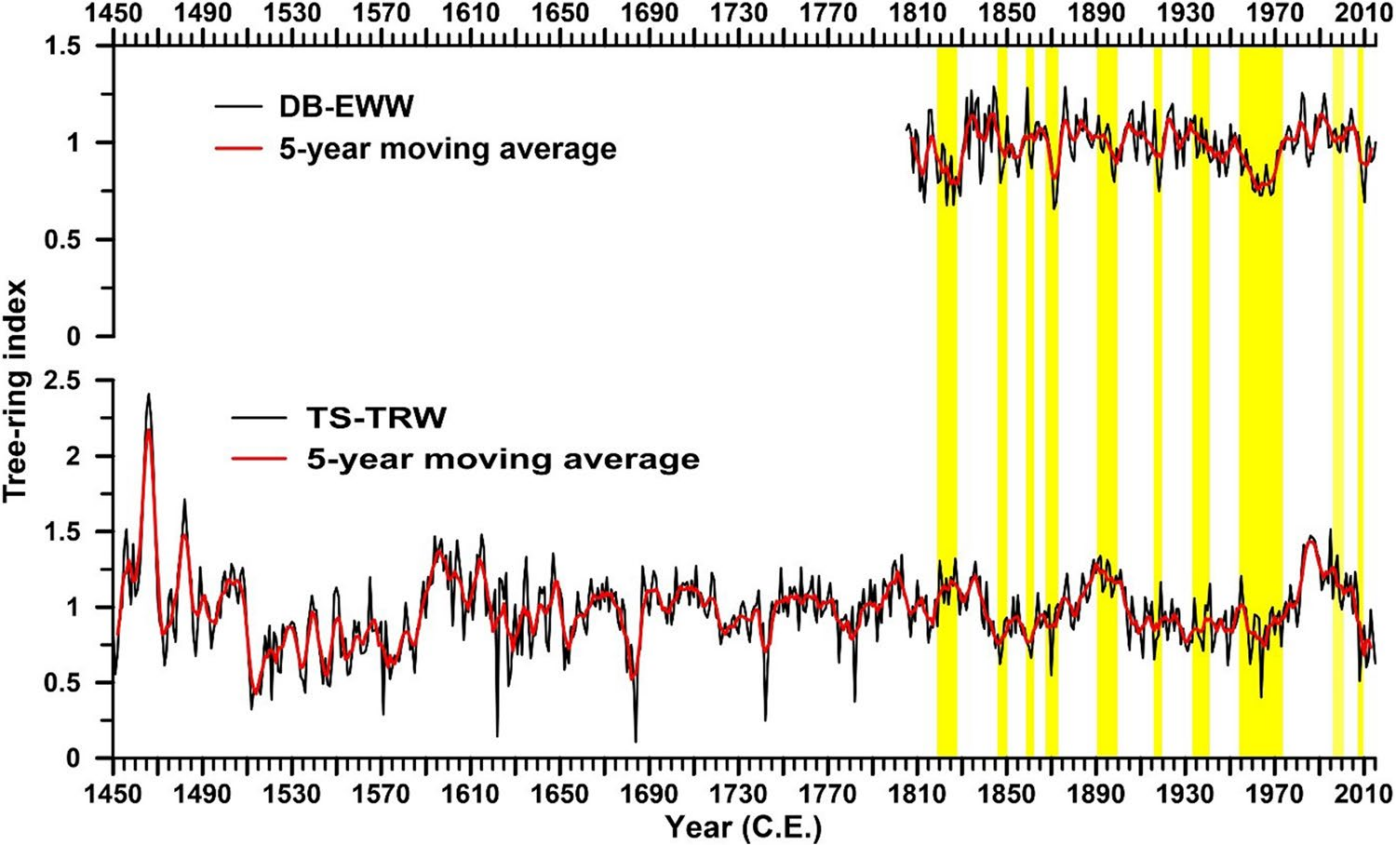
Earlywood width (DB-EWW) chronology of *Q. brantii* from Deh-Braftab (DB) site and tree-ring width (TS-TRW) chronology of *C. sempervirens* from Tang Soulak (TS) site. Vertical yellow columns indicate common years/periods of reduced growth between the site chronologies

DB-EWW (1805–2015) and TS-TRW chronologies (1450–2015) from the southern Zagros Mountains and their statistical characteristics are shown in Fig. 2 and Table 1. For the analyses of DB-LWW, please refer to Sect. 1 of the supplementary file. The values of first-order autocorrelation (AC1) for *Q. brantii* and *C. sempervirens* both indicate a significant influence of the climate of previous year on the current tree rings (Table 1). *C. sempervirens* shows higher mean sensitivity than *Q. brantii*, and EPS threshold (0.85) was reached after 1860 and 1560 C.E. for DB-EWW (6 trees) and TS-TRW (8 trees) chronologies, respectively. DB-EWW chronology shows similarities with TS-TRW chronology over the common period (1805–2015; Fig. 2), and DB-EWW (*r* = 0.48) showed stronger mean inter-series correlation than TS-TRW (*r* = 0.40).

**Table 1:** Statistical characteristics of DB-EWW and TS-TRW chronologies and calibration and leave-one-out verification models. AC1, first-order autocorrelation; MS, mean sensitivity; SNR, signal-to-noise ratio; SD, standard deviation, Rbar, mean inter-series correlation; EPS, expressed population signal; r, correlation coefficient; r1, correlation coefficient between observed data and the leave-one-out derived estimates; RE, reduction of error; PRESS, prediction residual error sum of squares; ST, sign test, which counts the number of agreement and disagreements between the observed data and estimated departures; DW, Durbin-Watson statistic. Two and three asterisks indicate significance at *p* < 0.01 and *p* < 0.001, respectively

Chronology statistics	DB-EWW chronology	TS-TRW chronology
Number of cores/trees	24/18	19/17
Period	1805–2015	1450–2015
Elevation (m a.s.l)	1810	1587
AC1	0.49	0.67
* MS*	0.19	0.26
* SNR*	4.79	2.39
* SD*	0.21	0.38
* Rbar*	0.48	0.40
* EPS* ≥ 0.85	1860	1560
Calibration and verification	DB-EWW chronology	TS-TRW chronology
Period	1987–2015	1987–2015
* r*	0.68***	0.59***
* R*^2^	0.46	0.35
* R*^2^_adj_	0.44	0.32
r1	0.60***	0.53**
* RE*	0.35	0.27
* PRESS*	62.2	7.4
* ST*	22/7**	20/9**
* DW*	1.90	2.02

**Fig. 3:**
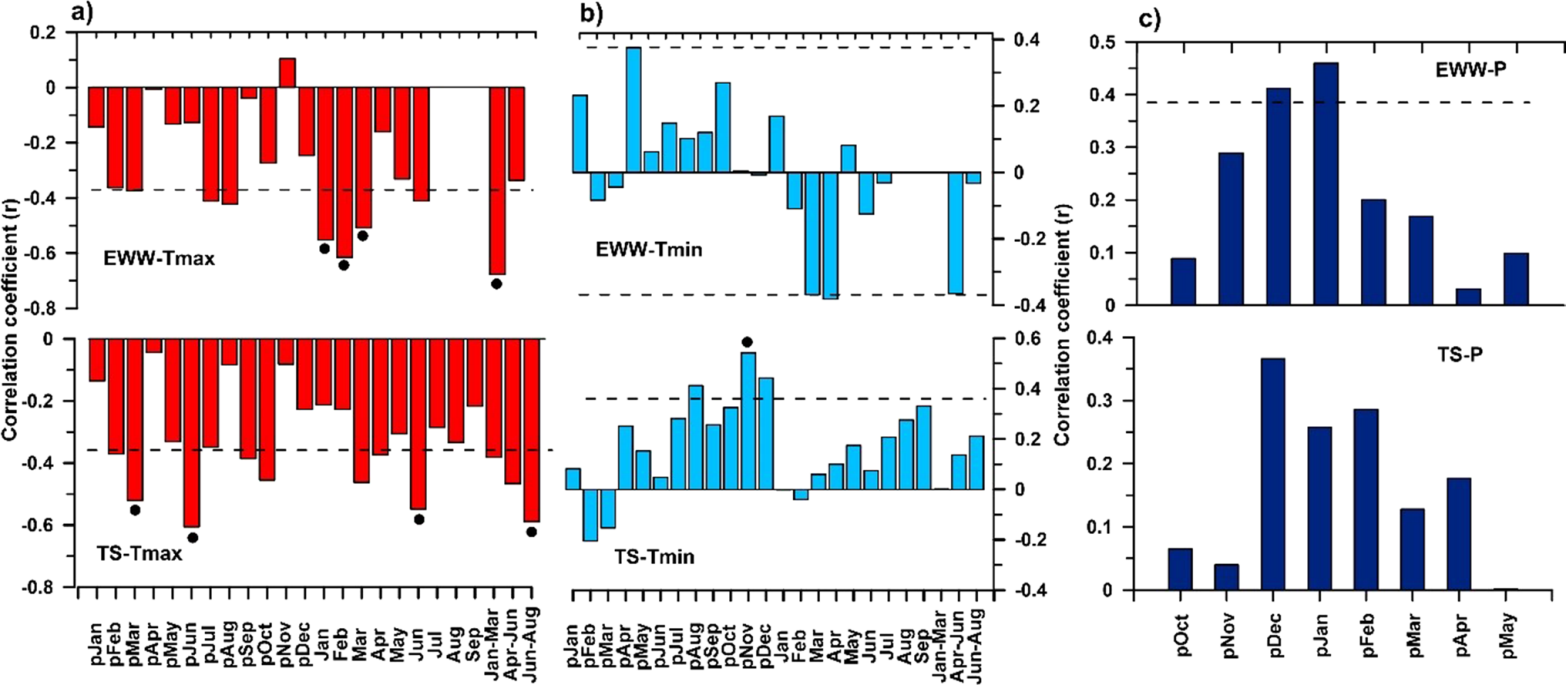
Pearson’s correlation coefficients for earlywood width (DB-EWW) chronology from *Q. brantii* and tree-ring width (TS-TRW) chronology from *C. sempervirens* with **a** mean monthly maximum temperatures (Tmax), **b** mean monthly minimum temperatures (Tmin) from the previous year January to current year September, and **c** monthly precipitation (P) from the previous year October to current year May over the period 1987–2015. Horizontal dashed black lines and the black solid circles indicate levels of significance at *p* < 0.05 and *p* < 0.01 levels, respectively

Evaluation of climate growth relationships revealed that Tmax from previous January to current year September, which has negative effects on the growth of *Q. brantii* and *C. sempervirens* (Fig. 3a), showed the highest negative correlations (*p* < 0.01) with DB-EWW during the current year (January to March). Tmax showed the highest negative correlations with the TS-TRW chronology during the previous year March and June and the current year June. It should be mentioned that Tmax showed strong negative correlations with the TS-TWR chronology during the previous and current year June. Correlations between seasonal Tmax and the TS-TRW chronology showed a strong significant negative correlation during June to August, while DB-EWW shows strong significant negative correlations with seasonal Tmax during January to March.

**Fig. 4:**
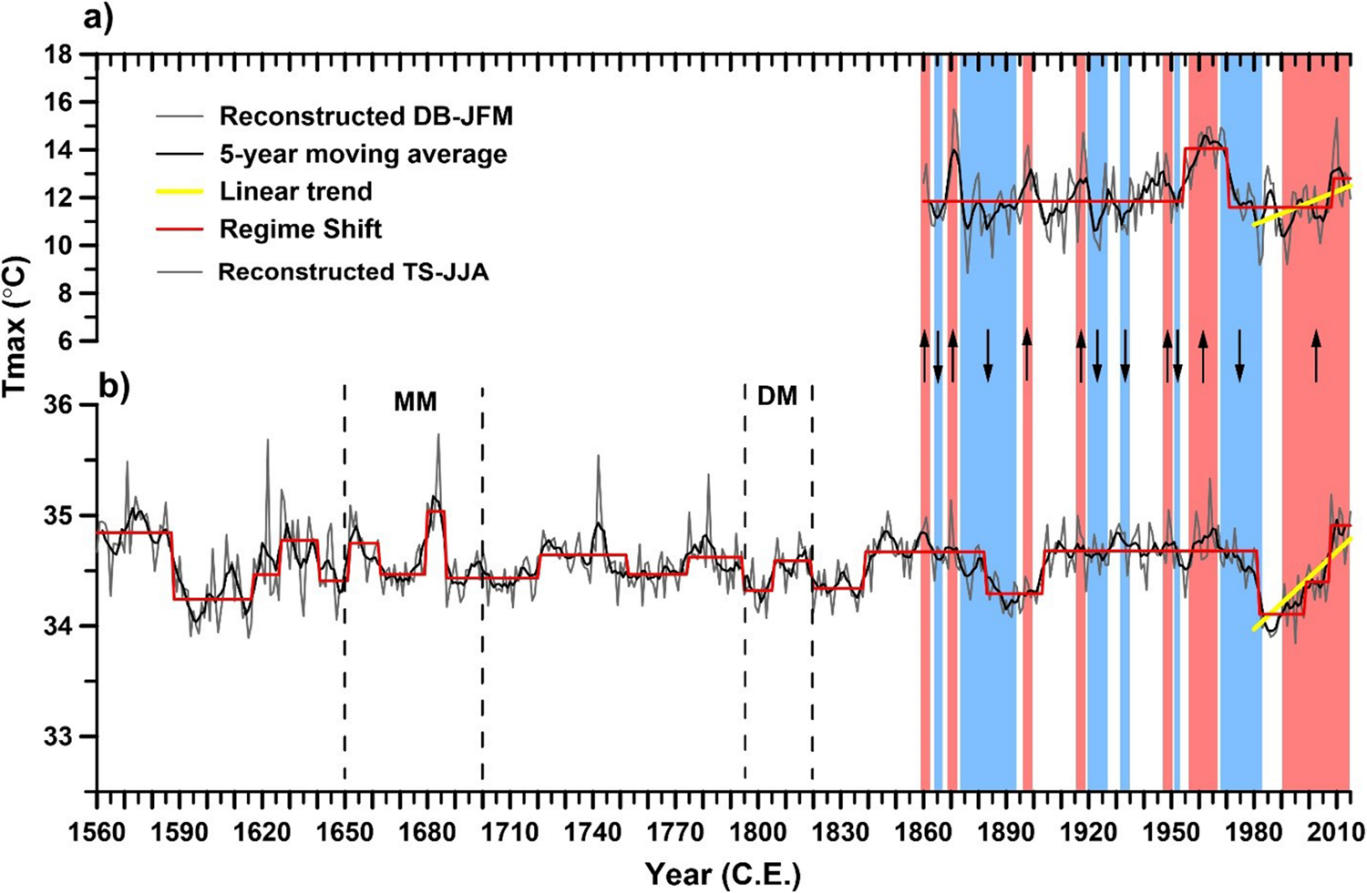
Mean maximum temperature reconstructions of **a** January–March (DB-JFM) and **b** June–August (TS-JJA) seasons in southwestern Iran. The vertical blue and red columns represent common cold and warm periods between the reconstructions, respectively. Bold red stair lines indicate regime shifts of the Tmax reconstructions. The regime shifts are statistically significant at *p* < 0.05. MM and DM indicate Maunder minimum and Dalton solar minimum, respectively

Tmin has negative relationships with DB-EWW chronology during the current growth year (Fig. 3b), whereas the relationship for the TS-TRW chronology is positive. In comparison to Tmin, Tmax showed stronger correlations with the chronologies in the southern Zagros Mountains. Correlations between the chronologies and monthly precipitation from the previous year October to the current year May (P) revealed positive effects of precipitation on oak and cypress trees in the region (Fig. 3c). Based on the highest correlations between DB-EWW and TS-TRW chronologies and the seasonal Tmax, we reconstructed DB-JFM (Fig. 4a) and TS-JJA (Fig. 4b) for the southern Zagros Mountains.

The linear regression models for the reconstruction of seasonal Tmax in DB-JFM and TS-JJA fulfill all statistical verification tests (Table 1) and confirm stability through time. The reconstruction validations are confirmed by sign test (ST) statistic and positive values of RE. The RE and PRESS values for DB-EWW and TS-TRW chronologies indicate the reliability of the derived Tmax reconstructions. The regression models account for 46% and 35% of the actual January–March and June–August Tmax variance during the calibration period 1987–2015, respectively.

DB-JFM and TS-JJA reconstructions cover 156 (1860–2015) and 456 (1560–2015) years, respectively (Fig. 2). A comparison between DB-JFM (*r* = 0.68, *p* < 0.01) and TS-JJA (*r* = 0.72, *p* < 0.01) reconstructions and instrumental climate data revealed high correlation between the time series over their common period 1987–2015 (Fig. S2a, b). Considerable annual and decadal temperature variability was retained in the summer and winter Tmax reconstructions for southwestern Iran. The winter and summer Tmax reconstructions showed very similar patterns (*r* = 0.32, *p* < 0.01), and most of the warm (42) and cold (45) years occurred simultaneously. The increasing trends of winter and summer Tmax started around 1965 after a relatively long cold period, though it should be mentioned that recent Tmax values in winter do not exceed previous maxima. The increasing trend of the reconstructed summer Tmax from 1980 to 2015 is stronger than that of winter Tmax.

Regime shift analysis showed two common significant warm (1955–1970 and 2008–2015) and one cold (1982–1998) periods in DB-JFM and TS-JJA reconstructions during the common period 1860–2015 (Fig. 4a, b). DB-JFM showed two regime shifts from low to high winter Tmax in 1955–1970 and 2008–2015 and one regime shift from high to low winter Tmax in 1970–2007 (Fig. 4a, b). The highest (0.89) and lowest (− 1.6) values of the regime shift for the reconstructed TS-JJA occurred in 2008 and 1982, respectively.

**Fig. 5:**
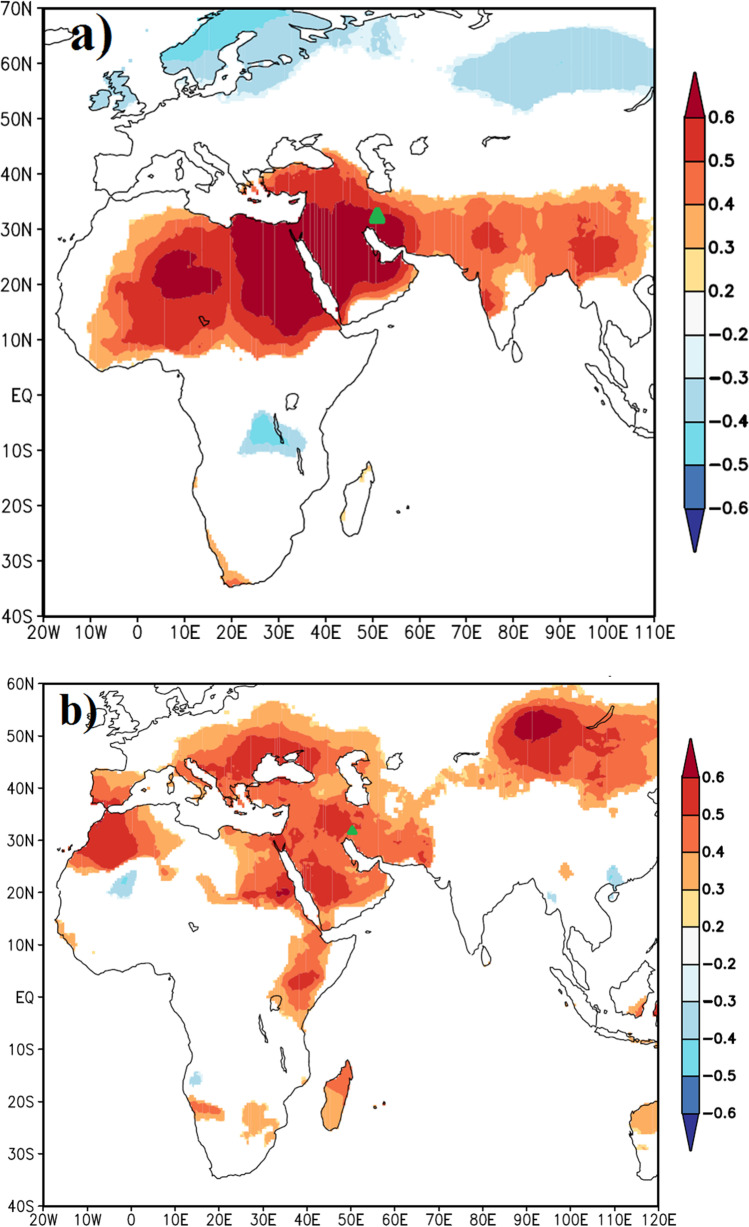
Spatial correlations between the reconstructed January–March (**a**) and June–August (**b**) mean monthly maximum temperatures and the regional gridded maximum temperature field at 0.5° intervals (CRU TS4.04; Jones and Harris, 2008) during the period 1980–2015. Green triangles denote the locations of the study sites

Spatial correlations between the reconstructed DB-JFM and regional gridded Tmax (CRU TS4.04; Jones and Harris 2008) showed highly significant positive correlations across a large area in North Africa, West Asia, the Indian peninsula, and southwestern China (Fig. 5a). There is an inverse relationship between the reconstructed winter (DB-JFM) Tmax and the regional gridded Tmax over the Scandinavian Peninsula and Siberia. Also, the reconstructed TS-JJA and the regional gridded Tmax showed strong relationships over a large area in West Asia, some parts of North and East Africa, Eastern Europe, and a large area in the northern Tibetan Plateau (Fig. 5b).

Correlations between the DB-JFM and TS-JJA reconstructions and climate indices revealed a significant negative correlation between NAO (1825–2015) and AO (1950–2015) and DB-JFM in the Zagros Mountains. Also, significant negative correlations were found between SNAO and PDO and the reconstructed TS-JJA. AMO showed positive relationships with the reconstructed DB-JFM and TS-JJA (Fig. S3a, b).

## Discussion

### Responses of *Q. brantii* and *C. sempervirens* to temperature variability

In this study, we used DB-EWW and TS-TRW to investigate the effects of Tmax and Tmin on regional dominant oak species and Mediterranean cypress trees in the Zagros Mountains, Iran. Clear boundaries of earlywood and latewood in *Q. brantii* make this xerophilous oak species very suitable for seasonal climate reconstructions in the Zagros Mountains (Arsalani et al. 2018b). The common years/periods of reduced growth of *Q. brantii* and *C. sempervirens* (Fig. 2) indicate that similar climate factors in the semi-arid region affect the radial growth of the broad-leaved as well as of the conifer species. As shown in Fig. 1, the largest amount of annual precipitation in the Zagros Mountains occurs during autumn and winter before the actual growing season (October to February). The values of AC1 (Table 1) confirm the impact of previous year’s climate conditions on the current year’s growth. Hence, both oak and cypress trees showed high correlations with previous year’s precipitation and temperatures (Fig. 3). The positive correlations of the oak and cypress chronologies with autumn precipitation confirm that storing carbohydrate at the end of the growing season is crucial for the wood production in the following growing season, supporting the relationship between previous year’s climate conditions and the current growth year.

In contrast to precipitation, Tmax showed strong negative impacts on tree growth of both investigated tree species. Hereby, the negative impact of Tmax on *C. sempervirens* is much higher than on *Q. brantii*, suggesting a higher sensitivity of evergreen cypress trees to warmer or drier conditions in the Zagros Mountains. Stronger negative relationships between DB-EWW and Tmax during the pre-growing and actual growing seasons indicate that Tmax is a main controlling factor for EWW of *Q. brantii.* This inverse relationship is further corroborated by negative relationships between earlywood vessel size parameters and mean monthly temperatures during the pre-growing and growing seasons (Arsalani et al. 2018b). Temperature is considered as the main climate factor for initiating tree growth after winter dormancy of the cambium, but high temperatures during the early growing season affect soil humidity by enhanced evaporation, leading to the formation of smaller earlywood vessels (Fonti and Garcia-Gonzalez 2008). Earlywood vessel size variability is among the main adaptation mechanisms that the native oak trees tolerate water stress. Despite the physiological properties and adaptation of *Q. brantii* as a native tree species, the current rapid increase in Tmax coupled to a decrease in soil water status has intensified the vulnerability of *Q. brantii* to climate change in the Zagros Mountains. Inverse relationships between temperature and previous oak chronologies (Arsalani et al. 2015, 2018a, 2018b) and *C. sempervirens* chronologies (Arsalani et al. 2021) during the pre-growing and growing seasons indicate that Tmax is the dominant climate factor driving tree growth in the Zagros Mountains, irrespective of the analyzed species.

Correlations with Tmin showed negative relationships with DB-EWW during the growing season. However, the relationship is only significant during February and March of the actual growth year, highlighting the negative impact of frost events on cambium dynamics and especially on the formation of earlywood vessels in the early growing season. Tmin also showed positive correlations with TS-TRW during the pre-growing and growing seasons, but the relationship is weaker in the early growing season. Severe frosts during the early growing season negatively influence ring formation and cause the formation of frost rings (Fig. S1c) in cypress, confirming the negative impacts of frosts on tree-ring formation in Mediterranean cypress during the early growing season.

### DB-JFM and TS-JJA reconstructions and large-scale temperature signals

Our two DB-JFM and TS-JJA reconstructions are significantly correlated (*r* = 0.30, *p* < 0.01) and show similar patterns during their common period (1860–2015). However, contrasting patterns between the two reconstructions occurred during 1900–1915 and 1930–1950 and 1984–1990. The TS-JJA reconstruction indicates that generally warmer conditions and the longest warm summer period occurred in the twentieth century. This warming trend was also reported by Köse et al. (2017) for Turkey. Additionally, TS-JJA reconstruction shows decreasing trends in the beginning of Maunder (1650–1700 C. E.) and Dalton (1796–1820 C. E.) minima of solar activity (Usoskin 2017). Furthermore, the significant cold summer regime shift in our TS-JJA reconstruction during 1820–38, which occurred after the Tambora volcanic eruption (1815) in Indonesia, coincides with a reported cooling period for the Tibetan Plateau between 1813 and 1827 (Cook et al. 2003; Li et al. 2011; Zhu et al. 2008, 2021; Huang et al. 2019). The persistent long-term cooling trends found in our temperature reconstructions in the 1890s (Fig. 4a, b) were also reported by Zhu et al. (2021) in the Karakoram/Pakistan. In line with the results of a tree-ring-based temperature reconstruction network from the Northern Hemisphere (Büntgen et al. 2021), we found for the Zagros Mountains strong warming trends since the 1980s in both, winter and summer Tmax reconstructions. Comparison between our DB-JFM and TS-JJA reconstructions and a Tmax reconstruction (May–June) in the Zagros Mountains (Arsalani et al. 2015) revealed that most of the reconstructed warm and cold periods occurred simultaneously (Fig. S4). Furthermore, the three seasonal Tmax reconstructions show increasing trends since 1986. Increasing trends of Tmax during 1980–2015 in the Zagros Mountains intensify evaporation from the shallow soils in open forests and tree canopy cover, thereby increasing drought stress in the region during the long dry summer.

Spatial correlations with regional gridded land surface Tmax revealed that our winter and summer Tmax reconstructions have high spatial representativeness. The similar patterns and strong spatial correlations between the reconstructed DB-JFM and regional gridded Tmax time series (CRU TS4.04; Jones and Harris 2008) in the subtropical regions from the North Africa to the Tibetan Plateau fit to the southward winterly shift of the westerlies, bringing the area under the influence of westerly wind belts (Fig. 5a). This region is mainly affected by mid-latitude westerlies during the winter, and this is confirmed by the similar patterns and strong relationships between the reconstructed DB-JFM and the regional Tmax. Subtropical high-pressure shifts to the higher latitudes in summer season. Accordingly, subtropical high-pressure cells are the main controlling factors of temperature variability in the dry summer season. It should be noted that descending air under subtropical high pressure is the main cause for temperature variability in arid Central Asia and Zagros Mountains, which is suggested by strong spatial correlations between the reconstructed summer Tmax in the Zagros Mountains and regional Tmax of arid Central Asia (Fig. 5b). The spatial correlations, which are similar to the results of Hughes et al. (2001), reflect the homogenous north hemispheric warming during the summer season in the regions which are outside of the Asian summer monsoon system. On the other hand, this pattern represents the northward shift of the westerlies during northern hemispheric summer, leading to higher influence of subtropical high-pressure cells in the Mediterranean region.

### Seasonal warm regime shifts and oak decline phenomenon

The highest regime shift in 1971 and 2008 and consecutive warm regime shifts at the end of twentieth century and at beginning of twenty-first century confirm the intensity of the Tmax variability and occurrence of record-breaking temperatures during the last decades in the Zagros Mountains. During the twentieth century shifts between the summer and winter reconstructions occurred simultaneously, confirming the effects of the global warming trend on temperatures in all seasons in the mountainous region of southwest Iran (Fig. 4a, b). An increase in Tmax during the winter may not only change precipitation type in the mountainous region (rain instead of snow) but could also increase population size of some invasive insects like oak leaf roller moth (*Tortrix viridana* L.), which is a common early season defoliator of oaks (Schroeder and Degen 2008) and causes considerable damage to the oak trees in the Zagros oak woodlands by feeding upon leaves (Ghobari et al. 2007; Banj Shafiei et al. 2011). In 2008, when the highest warm regime shift occurred, the oak decline phenomenon expanded epidemically (spread rapidly and covered large areas) in the Zagros oak woodlands. In this year, Iran experienced one of its most severe droughts, and all climate stations in the Zagros Mountains showed increased Tmax in winter (January–March) as well as in summer (June–August). It was reported that about 1 million ha of the oak woodlands were affected by this insect outbreak from 2008 to 2014 (Pourhashemi et al. 2017). It should be noted that *Biscogniauxia mediterranea*, which is responsible for most frequent disease of cork oak in the Mediterranean basin (Yangui et al. 2019), was detected as the main cause for the “charcoal disease” among *Q. brantii* trees throughout the Zagros Mountains (Mirabolfathy 2013). The simultaneous occurrence of fungal pathogen attacks and outbreak of oak leaf roller moth (*Tortrix viridana* L.) with the warm regime shifts and rapid increasing trends in summer and winter Tmax highlight the strong impacts of the ongoing climate change on the oak woodlands and fragile ecosystems in the Zagros Mountains.

### Relationships between seasonal Tmax reconstructions and large-scale teleconnection patterns

As a dominant mode of the Atlantic’s sector climate variability, NAO controls heat and moisture fluxes from the Atlantic Ocean into the Mediterranean region (Türkes 1996) and changes in Atlantic/Mediterranean sea surface temperatures (SSTs) and Atlantic westerly heat/moisture transport influencing the Middle East climate (Cullen et al. 2002; Kahya 2011). The influence of the Pacific Ocean’s and the Atlantic Ocean’s oscillations on Iran’s climate has been analyzed previously (Nazemosadat et al. 2015; Rezaei and Gurdak 2020; Arsalani et al. 2021). Significant negative correlations between NAO and our DB-JFM reconstruction (Fig. S3a) confirm that during positive phases of the NAO, the southern Zagros Mountains experience lower winter temperatures. During positive phases of NAO, cooler conditions prevail over the Mediterranean region (Scaife et al. 2008). Furthermore, significant negative correlations between NAO and winter surface air temperatures were reported for 53% of 51 climate stations in Iran (Ghasemi and Khalili 2006), including all stations located in the Zagros Mountains (Masih et al. 2011). Below and above normal winter temperatures were also reported in Iran during the positive and negative AO phases, respectively (Ghasemi and Khalili 2006). During the negative AO phase, westerly winds which originate from the warm Atlantic regions cause positive temperature anomalies in Iran, whereas the positive AO phase which is accompanied by northerly winds allows arctic air masses to move into the country, resulting in below-normal temperatures in winter over Iran (Ghasemi and Khalili 2006). The significant negative correlation between NAO and AO and DB-JFM (Fig. S3a) is further supported by inverse relationship between DB-JFM and the regional gridded Tmax over the Scandinavian Peninsula (Fig. 5a), highlighting the effects of the climate indices on temperature anomalies in the Zagros Mountains. The negative relationships between PDO and our seasonal Tmax reconstructions (Fig. S3b) are in line with the results of Goudarzi et al. (2017), who confirm the strong negative correlation between PDO and June–August Tmax of Karaj climate station near the capital Tehran. The AMO is a major mode of SST variations in the Atlantic Ocean. Positive correlations with our seasonal Tmax reconstructions confirm that during the warm (positive) phase of AMO, Tmax increase in the southern Zagros Mountains. This finding is supported by the results of Ahmadi et al. (2019) and Abbasi and Maleki (2017), who concluded that monthly mean air temperatures of Iran tend to increase during the warm (positive) phase of AMO. These correlations with the effective climate indices indicate that the variability of winter and summer temperatures in the Zagros Mountains is linked to changes in atmospheric pressure patterns of the subtropical high-pressure belt during the summer and of the westerly wind belt during the winter.

## Conclusions

In this study, we presented two new reconstructions of winter and summer Tmax based on DB-EWW (1860–2015) and TS–TRW (1560–2015) in the Zagros Mountains, Iran. Tmax in the winter and summer have strong negative impacts on the deciduous (*Q. brantii*) and coniferous (*C. sempervirens*) trees in the semi-arid region. Abrupt increases in winter Tmax (January–March), which strongly affect the earlywood part of *Q. brantii* tree rings, played an important role in stimulating outbreaks of invasive insects and fungal pathogen attacks in the Zagros oak woodlands during the last years (2008–2015), highlighting the vulnerability of the xerophilous oak species to ecological effects of global warming.

Our summer and winter Tmax reconstructions show high spatial representativeness, making the seasonal reconstructions more valuable in serving as a benchmark for the temperature history in the Middle East and eastern Mediterranean regions, where little information about climate history is currently available.

The most persistent warm summer regime shift in the twentieth century and the two consecutive warm summer regime shifts from 2000 to 2015 in the seasonal Tmax reconstruction confirm the consequences of global warming in the Zagros Mountains. Similar trends of winter and summer Tmax reconstructions during the last decades indicate the intensive effects of climate change on seasonal Tmax in this semi-arid region. Based on the abrupt changes in maximum temperatures, sustainable and adaptation plans mitigating the negative impacts of extreme maximum temperatures on the broad-leaved and evergreen species in the Zagros Mountains are urgently needed.

## Supplementary information

Below is the link to the electronic supplementary material.

Supplementary file1 (DOCX 2352 KB)
