# Supplementary material for: Tree-ring-based seasonal temperature reconstructions and ecological implications of recent warming on oak forest health in the Zagros Mountains, Iran
Source: Int J Biometeorol. 2022 Oct 10;66(12):2553–65. doi: 10.1007/s00484-022-02380-5 (PMC9684230; doi:10.1007/s00484-022-02380-5)
Supplement: Supplementary file 1 — Supplementary file1 (DOCX 2352 KB) [file 484_2022_2380_MOESM1_ESM.docx]

**Supplementary Material:**

International Journal of Biometeorology

**Tree-ring based seasonal temperature reconstructions and ecological implications of recent warming on oak forest health in the Zagros Mountains, Iran**

**Mohsen Arsalani ^1^,*, Jussi Grießinger ^1^, Achim** [**Bräuning**](https://www.sciencedirect.com/science/article/abs/pii/S003101822100081X#!) **^1^**

^1^ Friedrich-Alexander-Universität Erlangen-Nürnberg (FAU), Institute of Geography, Wetterkreuz 15, 91058 Erlangen, Germany

*) Corresponding author; [Mohsen.arsalani@fau.de](mailto:Mohsen.arsalani@fau.de). Tel: +49 9131 85-22920. Fax: +49 9131 85-22013

[jussi.griessinger@fau.de](mailto:jussi.griessinger@fau.de)

[achim.braeuning@fau.de](mailto:achim.braeuning@fau.de)


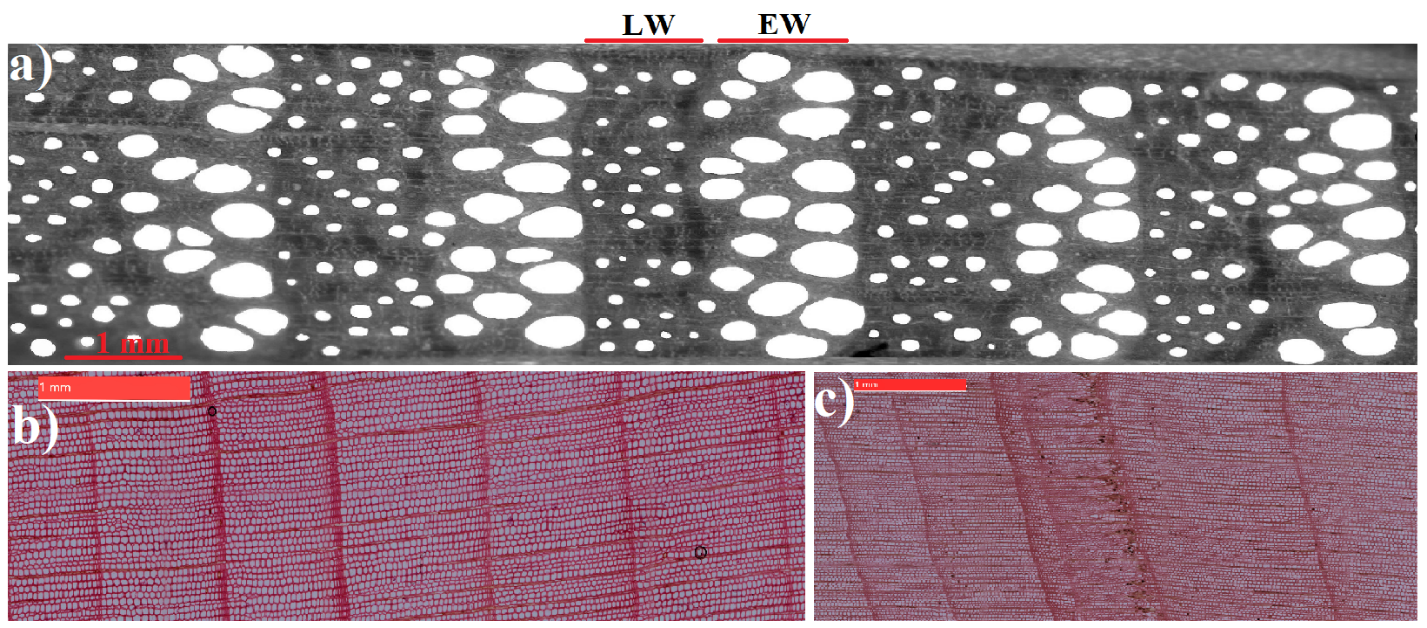


**Figure S1:** Surface image of *Q. brantii* (a) and micro section of *C. sempervirens* (b, c). C) Illustrates a frost ring in *C. sempervirens* wood from Tang Soulak site. The abbreviations annotated in the upper part of the surface image (a) indicate earlywood (EW) and latewood (LW) parts of a *Q. brantii* annual tree ring from Deh-Braftab site. The red bars in images b and c represent 1 mm


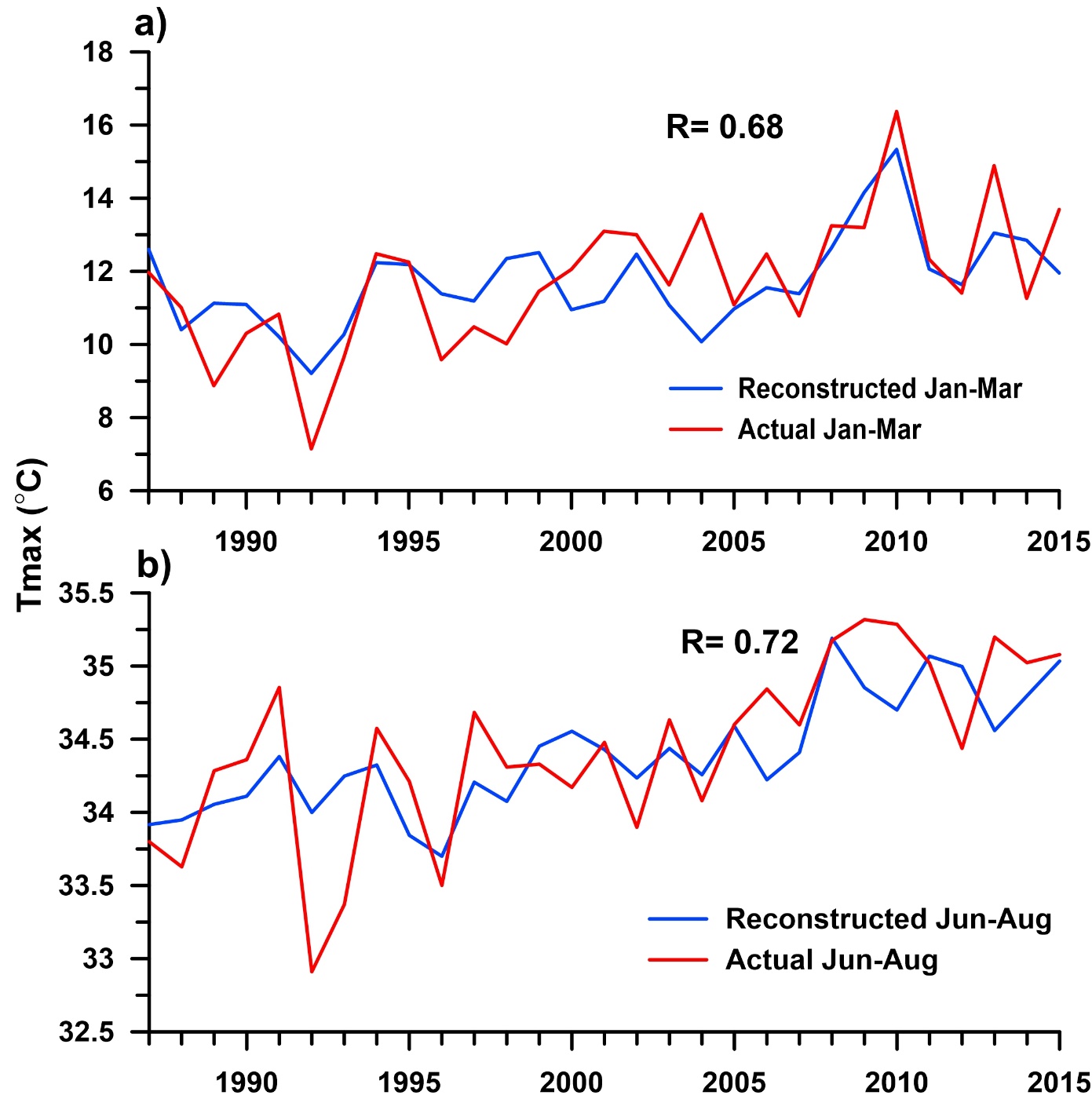


**Figure S2:** Actual and reconstructed January-March (a) and June-August (b) mean monthly maximum temperatures during the common period 1987−2015. R indicates the correlation coefficients between the actual and reconstructed maximum temperatures


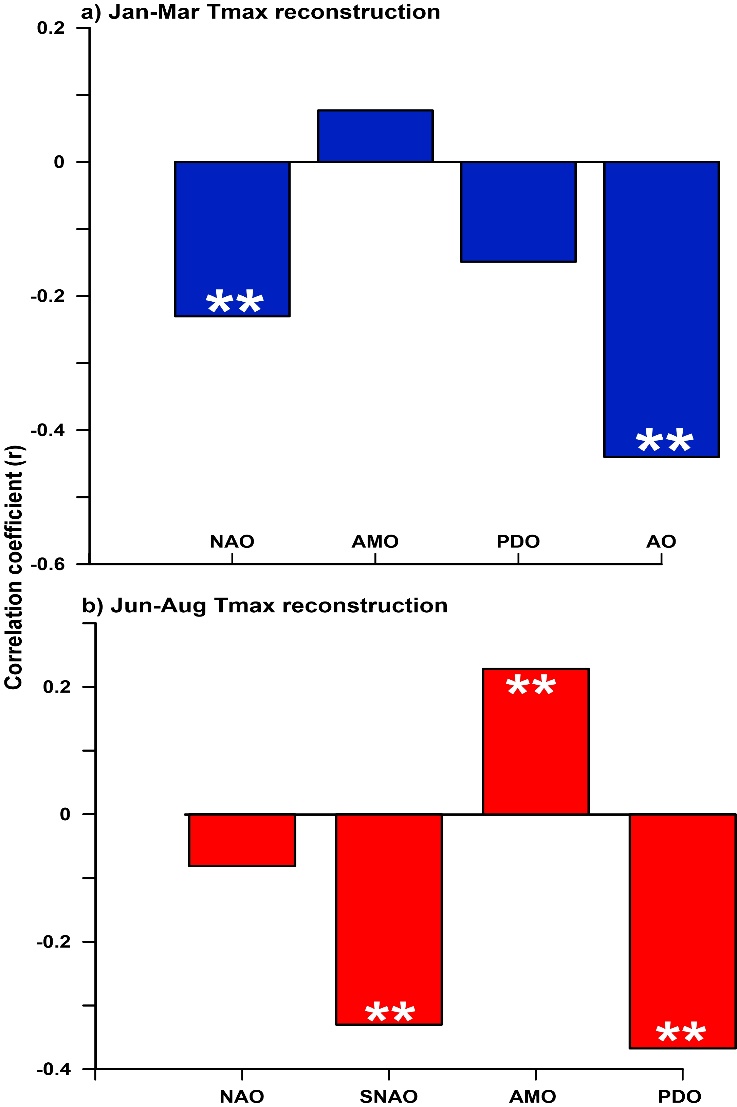


**Figure S3:** Correlation coefficients between the reconstructed January-March (a) and June-August (b) Tmax and the North Atlantic Oscillation (NAO; 1825-2015), the Atlantic Multidecadal Oscillation (AMO; 1880-2015), the Pacific Decadal Oscillation (PDO; 1911-2015), the Arctic Oscillation (AO; 1950-2015) and the Summer North Atlantic Oscillation (SNAO; 1948-2015). Two asterisks indicate significance at p < 0.01


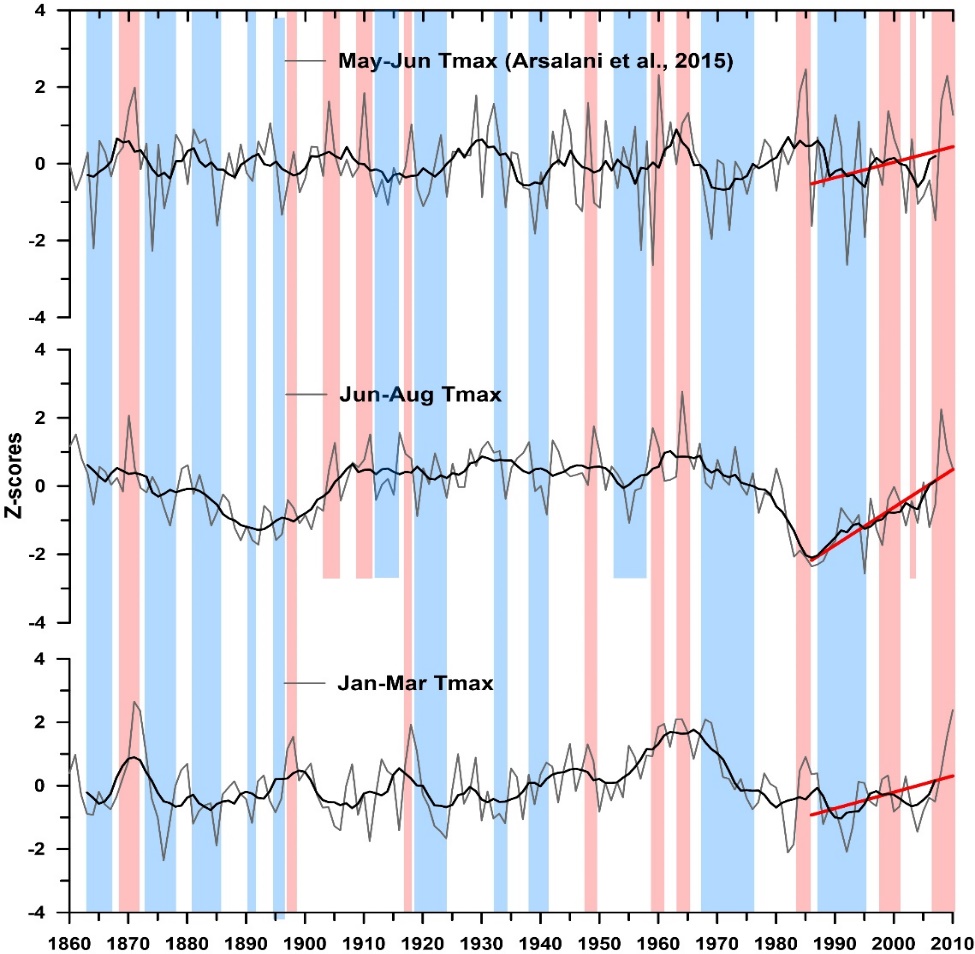


**Figure S4:** Comparison of reconstructed January-March and June-August Tmax in the southern Zagros Mountains with the reconstructed May-June Tmax in the central Zagros Mountains (Arsalani et al. 2015) during the common period 1860-2010. The solid black lines represent 7-year moving averages. The vertical blue and red columns represent common cold and warm periods, respectively. The solid red lines indicate linear trends from 1986 to 2010

**Section 1: DB-LWW analyses**

**
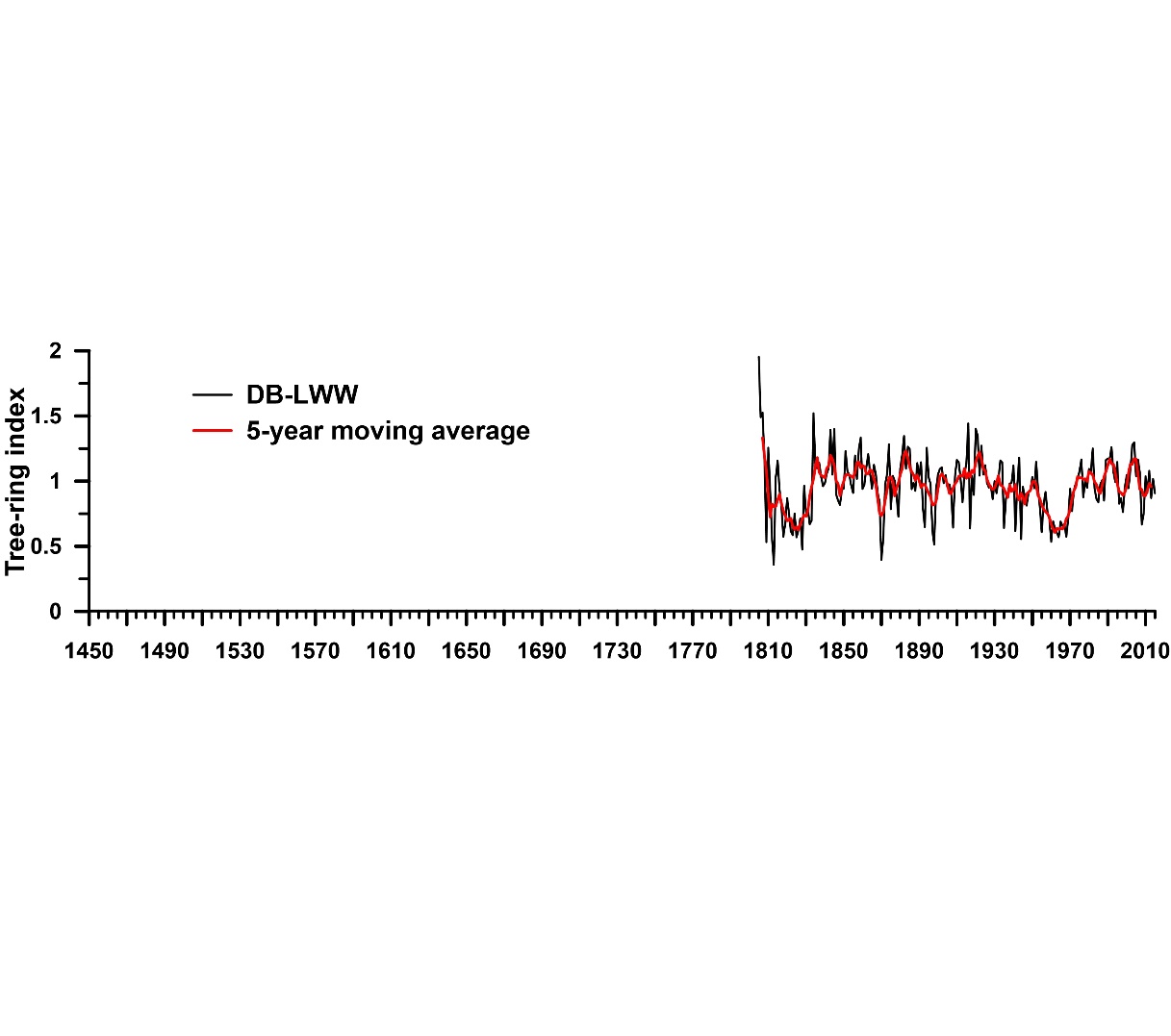
**

Latewood width (DB-LWW) chronology of *Q. brantii* from Deh-Braftab (DB) site.


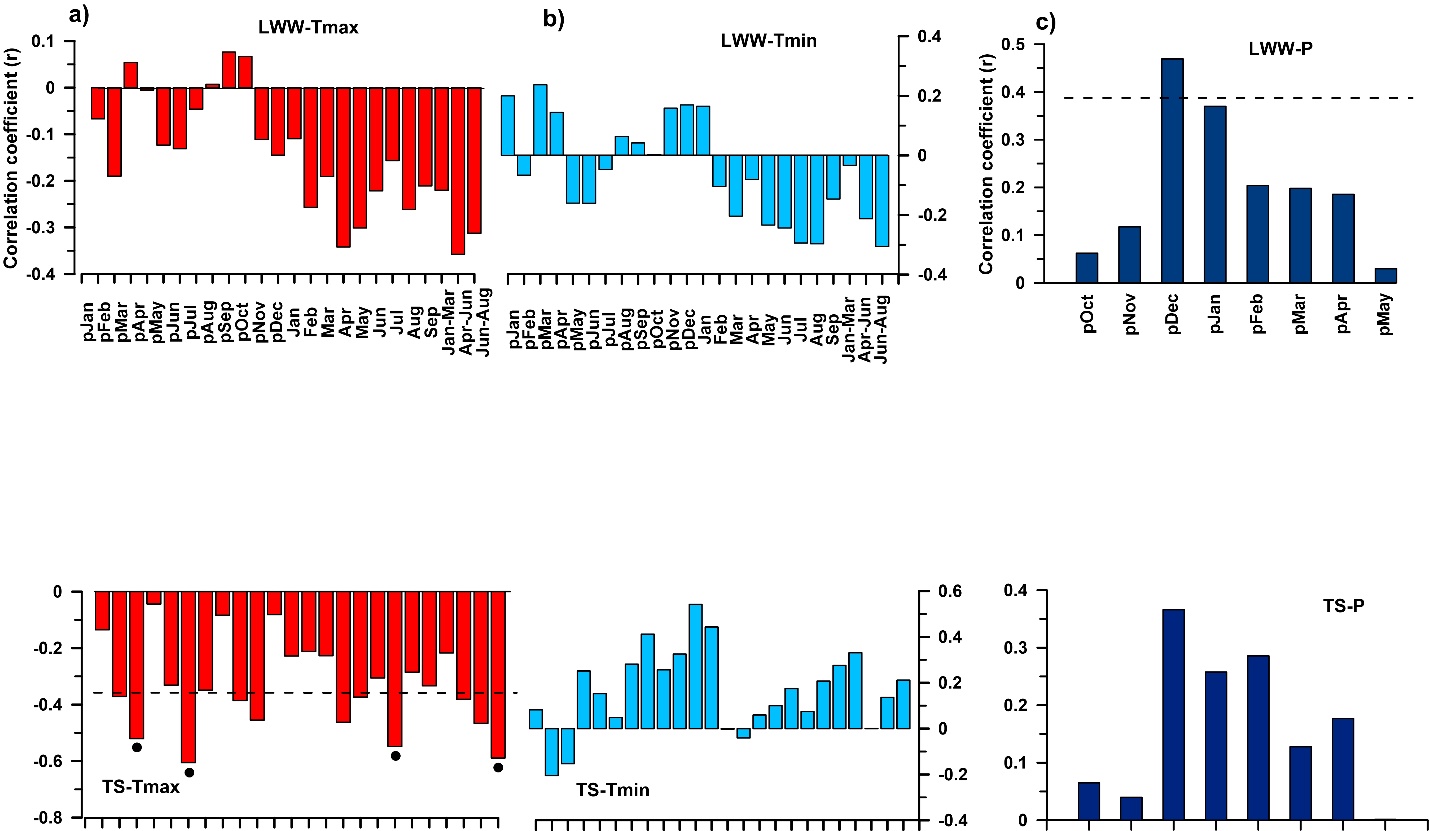


Pearson’s correlation coefficients for latewood width (DB-LWW) chronology from *Q. brantii* with a) mean monthly maximum temperatures (Tmax) and b) mean monthly minimum temperatures (Tmin) from the previous year January to current year September and c) monthly precipitation (P) from the previous year October to current year May over the period 1987-2015. Horizontal dashed black line indicates levels of significance at p < 0.05

Statistical characteristics of DB-LWW chronology. AC1, first-order autocorrelation; MS, mean sensitivity; SNR, signal-to-noise ratio; SD, standard deviation, Rbar, mean inter-series correlation; EPS, expressed population signal

| Chronology statistics | DB-LWW chronology |
| --- | --- |
| Number of cores/trees | 24/18 |
| Period | 1805-2015 |
| AC1 | 0.43 |
| MS | 0.34 |
| SNR | 3.45 |
| SD | 0.51 |
| Rbar | 0.39 |
| EPS≥0.85 | 1860 |
